# *Brucella abortus ure2 *region contains an acid-activated urea transporter and a nickel transport system

**DOI:** 10.1186/1471-2180-10-107

**Published:** 2010-04-10

**Authors:** Félix J Sangari, Ana M Cayón, Asunción Seoane, Juan M García-Lobo

**Affiliations:** 1 Departamento de Biología Molecular, Universidad de Cantabria, and Instituto de Biomedicina y Biotecnología de Cantabria (IBBTEC), UC-CSIC-IDICAN, Santander, Spain

## Abstract

**Background:**

Urease is a virulence factor that plays a role in the resistance of *Brucella *to low pH conditions, both *in vivo *and *in vitro*. *Brucella *contains two separate urease gene clusters, *ure1 *and *ure2*. Although only *ure1 *codes for an active urease, *ure2 *is also transcribed, but its contribution to *Brucella *biology is unknown.

**Results:**

Re-examination of the *ure2 *locus showed that the operon includes five genes downstream of *ureABCEFGDT *that are orthologs to a *nikKMLQO *cluster encoding an ECF-type transport system for nickel. *ureT *and *nikO *mutants were constructed and analyzed for urease activity and acid resistance. A non-polar *ureT *mutant was unaffected in urease activity at neutral pH but showed a significantly decreased activity at acidic pH. It also showed a decreased survival rate to pH 2 at low concentration of urea when compared to the wild type. The *nikO *mutant had decreased urease activity and acid resistance at all urea concentrations tested, and this phenotype could be reverted by the addition of nickel to the growth medium.

**Conclusions:**

Based on these results, we concluded that the operon *ure2 *codes for an acid-activated urea transporter and a nickel transporter necessary for the maximal activity of the urease whose structural subunits are encoded exclusively by the genes in the *ure1 *operon.

## Background

Urease catalyzes the chemical hydrolysis of the urea molecule into CO_2 _and ammonia. These equilibrate in water causing a rise of the pH of the medium. Accordingly, bacterial ureases serve two main purposes: to neutralize acidic conditions, and to provide a source of assimilable nitrogen.

Pathogenic bacteria exploit urease activity in different ways along the infectious process. In *Brucella *spp, as well as in *Helicobacter pylori*, *Klebsiella *and *Yersinia*, urease allows bacteria to survive the acidic conditions encountered in the stomach during the gastrointestinal infection [1-5]. The role of bacterial ureases in infectious disease has been recently reviewed [6].

Ureases are complex enzymes generally composed of three structural subunits (UreABC). To assemble a functional urease, the cooperation of several accessory proteins is required (UreEFGD) and, as a consequence, large gene clusters are needed to encode for functional ureases. *Brucella *contains two urease operons, both located in chromosome I. The *Brucella ure1 *operon contains the genes *ureDABCEFG*, and the *Brucella ure2 *locus shows the structure *ureABCEFGDT *[1]. The last gene of *ure2*, *ureT*, encodes a putative urea transporter homologous to Yut from *Yersinia pseudotuberculosis *[7]. Most *Brucella *species show a strong urease activity, derived from *ure1 *but not from *ure2*, and this activity is responsible for the ability of *Brucella *to survive stomachal transit and to establish a systemic infection [1,2]. *B. ovis *is not able to infect the host by the gastrointestinal route, a fact that has been linked to its lack of urease activity [8]. Furthermore, purification and characterization of urease from *B. suis *showed the presence of urease subunits from *ure1 *but not from *ure2 *[9]. Strikingly, *ure2 *genes are transcribed *in vivo *[1,2], suggesting that they play a role in *Brucella*.

Urease is one of the few enzymes known to contain nickel atoms in their active centers [10]. Because of this, the bacteria needs nickel uptake systems and a mechanism to incorporate the metal into the active center of the enzymes. Transition metal atoms are toxic and they cannot be free in the bacterial cytoplasm. Nickel should be delivered from the transport systems to chaperones that store the metal until needed for assembly. Chaperones and folding-assisting proteins are encoded by the urease accessory genes *ureDEFG *that form part of both *Brucella *urease operons. High affinity nickel transport systems of bacteria fall into several categories: the ATP-binding cassette (ABC) systems represented by NikABCDE of *E. coli *[11], the newly described Energy-Coupling Factor (ECF) transporters like NikMNQO [12] and secondary transporters from different families that include NiCoT [13], UreH [14], and HupE/UreJ [14,15]. The ECF transporter NickMNQO consist of substrate-specific module (S components, NikMN), which are integral membrane proteins, and an energy-coupling module that contains an ATPase typical of the ATP binding cassette (ABC) superfamily (A component, NikO) and a characteristic transmembrane protein (T component, NikQ). It may contain additional components like NikL, which is an integral membrane protein, or NikK, a periplasmic protein [12,16]. In *Brucella suis*, a nickel ABC transporter coded by the *nikABCDE *gene cluster has been identified. This gene cluster has been shown to contribute towards the urease activity of the bacteria when Ni ions are chelated with EDTA in the growth medium, but not in control media without EDTA. This implies, as noted by the authors, that there is at least another functional nickel transport system in this bacteria [17].

Urease activity is also dependent on the supply of urea. There are at least three urea uptake systems in bacteria. The ABC-type urea transporter is energy-dependent and requires ATP to transport urea across the cytoplasmic membrane. The other two urea transporters, Yut and UreI, are energy-independent and appear to be channel-like structures that allow urea to enter the cytoplasm through a pore powered by a favorable concentration gradient that is maintained by rapid hydrolysis of the incoming urea by intrabacterial ureases. The recent determination of the crystal structure of the *Desulfovibrio vulgaris *urea transporter [18] confirms the existence of an unoccluded channel for urea, with a 'molecular coin-slot' mechanism that allows urea to pass through the transporter in preference to other small molecules. This selective filter consists of two hydrophobic slots in series, just wide enough to permit the coin-shaped urea molecule to enter. Each slot is formed by two phenylalanine amino-acid residues, an "oxygen ladder" lying along one side of the slot, and several hydrophobic phenylalanine and leucine residues lining the pore opposite to each of the oxygen ladders. The oxygen ladder provides electrostatic interactions for the urea molecules, helping to extract them from the aqueous environment outside the channel. The urea channels are composed of different numbers of membrane-spanning helices (six for *Helicobacter *UreI, ten for *Yersinia *Yut), that in the case of Yut and UreT form two repeated domains linked by a large periplasmic loop. However, the most important difference between UreI and Yut is their response to acidic pH. While Yut shows similar activity at a range of different pH [7], UreI shows a 6- to 10-fold activation at pH 5.0 compared to pH 7.5 [19]. The presence of protonable residues (histidines or carboxylates) in the periplasmic loops of UreI seems to be responsible for this activation, and the mechanism of proton-gating presumably is a conformational change in the membrane domains of UreI induced by a change in the state of protonation of those residues [20]. Both nickel and urea transport systems are required in order to reach maximum levels of urease activity.

The evidence presented here shows that the urease operon *ure2 *includes genes for the transport of urea and nickel, and that these genes are expressed and active, contributing to urease activity and to resistance to the acidic conditions present in the oral route of infection.

## Results

### Evidence of transcription and redefinition of the *ure2 *operon of *Brucella abortus *2308

**Figure 1:**
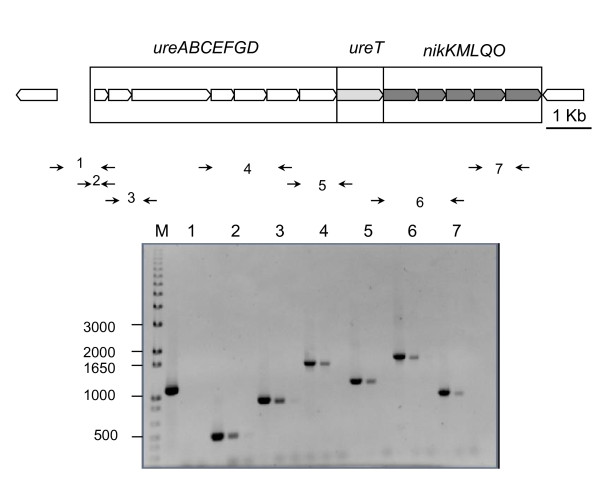
**Evidence of transcription and redefinition of the *ure2 *operon of *Brucella abortus *2308**. The map on top of the figure shows the *ure2 *region of the large chromosome of *Brucella abortus *2308. Below the map the arrows indicate primers designed to check transcription of the region. For each pair of primers marked with a number, three separate PCR reactions were performed: a positive control using genomic DNA as template; a test reaction using cDNA as template, and a control using RNA as template. M, 1 Kb Plus DNA ladder.

We have previously reported that the *Brucella *urease operon *ure2 *did not contribute to the urease activity of the bacteria [1]. The *ure2 *operon of *Brucella abortus *2308 was considered to be composed of eight genes *ureABCEFGDT *(BAB1_1376-1383). A re-evaluation of the chromosomal region suggested that some genes immediately downstream of *ureT *could be part of the same operon, because: 1) the distance between *ureT *and the contiguous gene *nikM *was only 26 bp, 2) there was a good ribosome binding site upstream the putative start codon of *nikM*, and 3) there was no obvious transcriptional terminator between the two genes. PCR amplification of reverse transcribed *Brucella *RNA using the pairs of primers indicated in Table 1 was conducted to assess the continuity of the transcript until we reached the first gene annotated on the opposite strand (BAB1_1389). Genomic DNA and total RNA were used as positive and negative controls, and the results are shown in Figure 1. Five additional genes (BAB1_1384-1388) were found to be cotranscribed with the first eight genes, and their functional gene annotation was performed using the SEED comparative genomics resource [21]. The proposed role of these genes (*nikKMLQO*) was to code for a nickel transport system belonging to the novel ECF class of modular transporters [12]. According to this classification, NikM would be the substrate-specific component, while NikQ and NikO would be the transmembrane and ATPase components, respectively, of the energizing module. NikK and NikL would be additional components. Their role in nickel transport is also supported by the genomic context, as urease is a nickel-containing enzyme, although we could not find other supporting evidence (e.g. NickR-binding sites in the region). The complete *ure2 *operon is thus composed of thirteen genes putatively involved in three different functions, namely urease production, urea transport, and nickel transport.

**Table 1:** Oligonucleotides

RT PCR		Gene set
RT_BAB1_1374_*Bam*HI.F	GGATCCACACGCGATTTCCTTTCATC	1

RT_*ureA2*_*Bam*HI.R	GGATCCCATCACCTCTTCGACGGTTT	1, 2

RT_BAB1_1375.F	AAGGTCCTGCCAGTACAACG	2

RT_*ureA*2.F	AAACCGTCGAAGAGGTGATG	3

RT_*ureC*2.R	CGCAGATCCTTCTCGATTTC	3

RT_*ureC*2.F	ACAGTCGATCTCGCTCAACC	4

RT_BAB1_1381.R	CTTGATAAGGATTGGCACGA	4

RT_BAB1_1381.F	ACCTGATCCGTGAAAACGTC	5

RT_BAB1_1383.R	GAAAGAACAGTCCCGTCAGC	5

RT_BAB1_1383.F	GGATACAACCAAGCCTGCAT	6

RT_BAB1_1386.R	GGCATTGCGGATGATAAGTT	6

RT_BAB1_1386.F	GCTTTTTCTCTGGGCCAAAT	7

RT_BAB1_1388.R	GACAGGGAAAGCTTGTCGAG	7

*ΔureT*		

U_BMEI0642_*Xba*I.F	TCTAGAGACCCAGACCATAACGCTTG	

U_BMEI0642_*Bam*HI.R	GGATCCCTGCCATGGAGGCCTCCT	

BMEI0642.F	AGGAGGCCTCCATGGCAGGGATCCCCTGAGCCTGATTTCTGGA	

D_BMEI0642_*Pst*I.R	CTGCAGGACCGATCCGTCATTGACAT	

*aphT*		

*aphT*.F	ATACTGCAGATTAGAAAAACTCATCG	

*aphT*.R	TCACACAGGAAACAGCTATG	

*ΔnikO*		

BAB1_1388 *XbaI*.R	ACGTTCTAGACAATATCTGCGTGCTCTCCA	

RT_BAB1_1388.R	GACAGGGAAAGCTTGTCGAG	

BAB1_1388 *Bgl*II.F	CTCGACAAGCTTTCCCTGTCAGATCTCCACCTGCATTATGTCGAG	

BAB1_1388 *Pst*I.R	ACGTCTGCAGCATTATCGATAGCGGCCTTG	

### Construction of chromosomal mutants in the *ure2 *operon

In order to analyze the impact of the *ure2 *genes on urease activity, we constructed three mutants as described in the Methods section: i) a polar mutant created by replacing part of *ureT *with a kanamycin resistance gene that has a transcriptional termination signal (*ΔureTp*), ii) a non-polar mutant lacking the *aph *transcriptional terminator, which only affects *ureT *function (*ΔureT*), and iii) a *ΔnikO *mutant, affecting the ATP binding protein of the putative nickel transport system encoded by *nikO*, the last gene of the operon, and predicted to have the biggest impact on the correct function of the transporter while still maintaining basal activity [16].

### Urease activity of the different *ure2 *mutants

**Figure 2:**
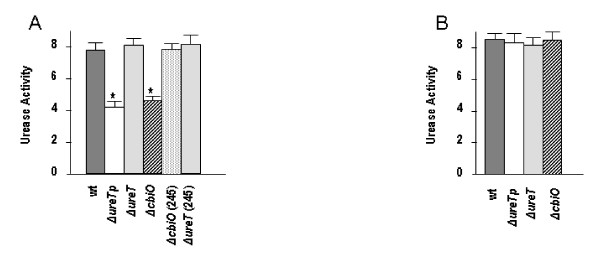
**Urease activity of *B. abortus *2308-derived strains**. Urease activity was determined in bacterial extracts obtained from the indicated strains and growth conditions, and expressed in μmol of NH3 min^-1 ^mg^-1 ^protein The experiments were performed by triplicate with three technical measures per replica. The data shown correspond to one representative experiment and the error bar indicates the standard deviation. An unpaired t-test was performed to determine if the urease activity of each mutant was significantly different than the corresponding wild type control. A. Protein extracts from cultures of the indicated strains grown in BB. B: Protein extracts from cultures grown in BB supplemented with 0.5 mM NiCl_2_. * indicates p < 0.05.

Urease activity was measured in crude protein extracts from the mutants and the wild type strain. The results in Figure 2A show that extracts of both the *ΔureTp *and *ΔnikO *mutants had their urease activity reduced to about 50% of the activity observed in the wild type strain 2308, while the urease activity was rather unaffected in the *ΔureT *mutant. To confirm that the observed effects were direct consequence of the introduced mutations, mutants were complemented with plasmids carrying either *ureT *or *nikO *genes. Mutants *ΔureTp *and *ΔnikO *were complemented by the *nikO *containing plasmid pFJS245 (Figure 2A), but no effect on urease activity was observed when pFJS243 (containing *ureT*) was used to complement the *ΔureTp*, *ΔureT*, or *ΔnikO *mutants (data not shown).

### Effect of nickel addition on urease activity of different *Brucella *strains

Nickel and cobalt are transition metals that can share the same bacterial import systems [16]. The genes *nikKMLQO*, currently annotated in the *Brucella *genomes as components of a cobalt transport system, are found downstream of the *ure2 *genes, and form part of the same operon, so we tested whether they were involved in the transport of nickel, which is essential for urease activity. The addition of an excess of nickel in the form of NiCl_2 _would supply the metal needed for urease assembly in spite of the inactivation of the *nik *transport system. We tested the urease activity of the different strains grown in the presence or absence of 0.5 mM NiCl_2 _in the culture medium. The results in Figure 2B indicate that the urease activity of all the mutants reverted to normal values when the culture medium was supplemented with nickel, thus confirming the suspected role of the products of the *nik *genes of the *ure2 *operon in nickel transport. These results are also a further evidence for the extension of the operon until the *nikO *gene; that is a polar *ureT *mutation has a lower urease activity than the corresponding non-polar mutant, and identical activity to that of the *nikO *mutant, suggesting that the observed phenotype is the result of a polar effect on the genes downstream of *ureT*.

### Effect of pH on urease activity in intact cells

**Figure 3:**
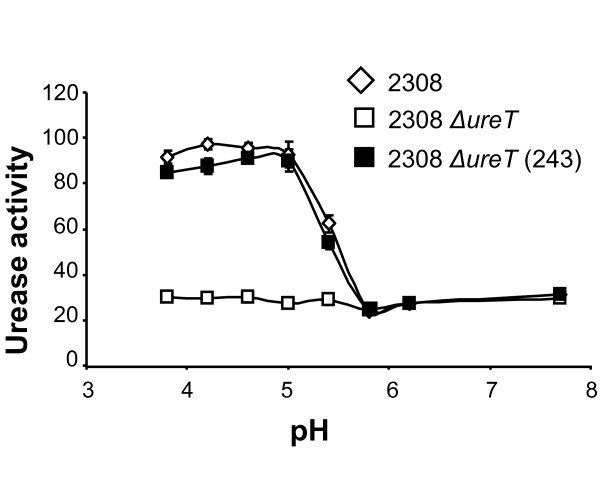
**pH dependency of urease activity in intact *Brucella *cells**. Intact cells were exposed to the indicated pH for 15 minutes, in buffer containing 5 mM urea and then urease activity determined, and expressed in pmol of NH_3 _min^-1 ^log10 cfu^-1 ^(diamond) 2308, (white square) 2308 *ΔureT*, (black square) 2308 *ΔureT *(pFJS243).

*Brucella *urease assayed *in vitro *shows a pH-dependent activity that is maximal at pH 7.3 [1]. When urease activity was assayed in intact *B. abortus *2308 cells, the activity was higher at low pH values and dropped to near one third as the pH of the medium reached a value of 6 (Figure 3). *ΔureT *intact cells showed very similar activity to wild type cells at pH values above 6, but they lost the acid-dependent induction of urease activity at lower pH values. The increased urease activity of the *ΔureT *mutant due to enhanced urea uptake at low pH could be restored by complementation with the *ureT *containing plasmid pFJS243.

### Effect of urea concentration on urease activity in intact cells

**Figure 4:**
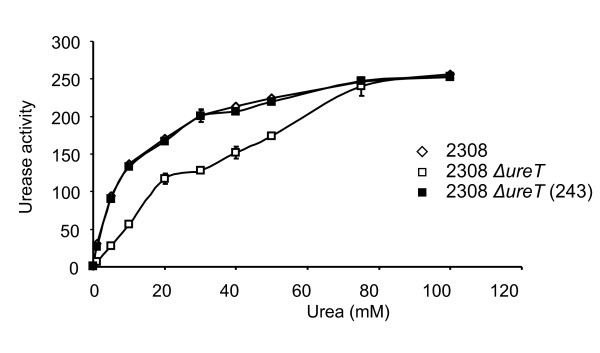
**Urease activity in a urea gradient**. Intact cells exposed to buffer pH 4.2 with increasing amounts of urea. (diamond) 2308, (white square) 2308 *ΔureT*, (black square) 2308 *ΔureT *(pFJS243).

As the observed results were consistent with UreT being a urea transporter, 2308, 2308 *ΔureT*, and 2308 *ΔureT *(pFJS243) were exposed for one hour to increasing concentrations of urea (pH 4.2). The urease activity of both the wild type and the complemented strains increased steadily with the available urea. However, the *ΔureT *mutant showed significantly lower activities at all the urea concentrations tested, except for 75 and 100 mM, where urease activity reached wild type levels (Figure 4), presumably because membrane diffussion surpasses carrier mediated transport at these urea concentrations.

### *In vitro *susceptibility of *Brucella *to acid pH

**Figure 5:**
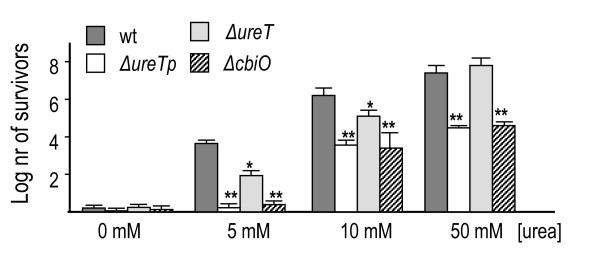
**Survival of *B. abortus *urease mutants to acid exposure**. Log n° of bacteria surviving an acid shock of 30 minutes at pH 2.0 in the presence of different amounts of urea. The arithmetic media from three separate experiments was plotted with standard deviations. An unpaired t-test was performed to determine if survival of each strain was significantly different than the corresponding wild type control. * indicates p < 0.05, ** p < 0.01.

It has been shown that under long (15 min) exposures to highly acidic environments (pH 2.0), urease activity in the presence of urea in the medium enables *Brucella *survival [1,2]. The *ΔureT *mutant showed a susceptibility to acid significantly higher than the wild type but lower than the *ΔureTp *and *nikO *mutants at low concentrations of urea (5-10 mM). At 50 mM urea the *ΔureT *mutant was as resistant as the parental strain, while the *ΔureTp *and *nikO *mutants remained significantly susceptible (Figure 5).

The susceptibility to low pH of the mutant *nikO *was completely reversed by complementing it with pFJS245 in *trans*. The mutant *ΔureTp *could not be complemented in this assay with either pFJS243 or pFJS245 (data not shown). However the acid sensitivity of both mutants could be compensated by the addition of NiCl_2 _to the growth medium (data not shown).

## Discussion and Conclusions

The presence of two operons encoding urease in the genome of *Brucella *had already been reported. Evidence from our laboratory and elsewhere [1,2,9] showed that only urease from *ure1 *contributed towards the urease activity of *Brucella*. Explanations based on a degeneration in the sequence of *ure2 *were ruled out and early evidence suggested that *ure2 *genes were transcribed [1,2]. Accordingly, we suggested that *ure2 *should have some function that ensures its conservation in the genome of *Brucella *[1].

In this work, we analyzed the transcription of the *ure2 *operon by RT-PCR, confirming that the *ure2 *genes are transcribed and that transcription goes beyond *ureT*, up to the gene *nikO *(BAB1_1388) (Figure 1).

While our RT-PCR experiment did not show a full-length transcript, it demonstrated the existence of messenger RNA molecules containing both *ureT *and *ureD *and also *ureT *and *nikM*. Furthermore, the introduction of a polar mutation in *ureT *had different effects than the introduction of a non polar mutation in the same gene, and the polar effects could be explained by the absence of activity of distal *nik *genes. Pooling this data, the most plausible explanation is that all the genes in the revised *ure2 *cluster form a single transcriptional unit that we have termed *ure2ACBEFGDTnikKMLQO*. We cannot rule out the possibility of secondary promoters existing in this region.

By compairing the mutant strains to the wild type progenitor we observed that there was no significant difference in urease activity between protein extracts from *B. abortus *2308 and the *ΔureT *mutant, but the analysis of urease activity in intact cells at different pH's revealed that, while the wild type strain showed a sharp increase in urease activity at pH values lower than 5.8, the activity of the *ΔureT *mutant remained unchanged. The amount of active urease in protein extracts from the *ΔureT *mutant was the same as that of the 2308 parental strain, indicating that urease biosynthesis was not affected. However UreT contributes towards urease activity in intact cells by facilitating the access of urea to the cytoplasm. Our results indicate that the urea transporter plays a role at low urea concentrations, equivalent to those encountered in host tissues. At higher concentrations, urea diffusion through the inner membrane probably compensates for the absence of the transporter. Remarkably, the activity of the transporter (measured as urease activity in this case) was pH-dependent. The activity observed at pH 5.8 or higher would be the result of urea diffusing through the inner membrane. That UreT is an acid-activated urea transporter is somewhat surprising, given that its closest homolog, Yut of *Y. enterocolitica*, is not pH-regulated [7], while the best known example of a proton-gated urea channel, UreI of the gastric pathogen *H. pylori *[20], shares a rather low amino acid sequence identity to UreT. The mechanism of proton-gating has been proposed to be a conformational change in the membrane domains of UreI induced by a change in the state of protonation of some residues (histidines or carboxylates) in the periplasmic loops. Modelling of UreT, UreI and Yut with TMRPres2D [22] revealed some differences between the protein sequences at the periplasmic loops. While UreI presents a total of fourteen protonable residues, Yut has only three, and UreT possesses seven (data not shown). The higher number of protonable residues of UreT could account for the differences found in acid activation between Yut and UreT. However, the mechanism of urea selectivity is probably the same, as a comparison with the crystal structure of the urea transporter of *D. vulgaris *shows that all the residues that form the pore are conserved (data not shown). The only one minor difference is that in one of the two urea slots present in UreT, one of the phenylalanines forming the slot is changed to leucine (L201F), and the corresponding leucine in the slot is changed to phenylalanine (F304L) (data not shown).

Since urea uptake is not pH regulated in *Yersinia *spp, the unrestricted entry of urea would alkalinize the cytoplasm to lethal levels. *Yersinia *has solved this problem by expressing a urease with an acidic pH-optimum, that has little or no activity at ~pH 8.0 [5]. *Brucella *urease has a pH optimum of 7.3, and although its activity is much lower at pH 8.0, it is still significant. In this case, the problem of lethal alkalinization is prevented by the existence of a pH-regulated urea transporter that reduces urea uptake to just the amount that diffuses through the inner membrane.

In contrast to the *ΔureT *mutant, mutants *ΔureTp *and *ΔnikO *showed around a 40% decrease in urease activity in cell extracts. Both phenotypes were reversed by complementation of the mutant strains with a *nikO*-containing plasmid or, alternatively, with high concentrations of nickel in the culture medium suggesting that the amount of active urease in these mutants was limited by nickel availability. Complementation of the urease activity of the *ΔureTp *mutant with the *nikO *plasmid was rather surprising if we consider that the mutant should be defective not only in *nikO *but also in the other *nik *genes. Furthermore, the susceptibity to low pH of the *ΔureTp *mutant was not complemented by the *nikO *gene *in trans*, suggesting that other factors may be implicated in the acid resistance phenotype of *Brucella*.

NikO is predicted to be the ATPase component of an ECF-type nickel transporter, and its mutation should abolish most of the activity of the transporter. There is another nickel transport system already described in *B. suis*, NikABCDE (10). *nikA *mutants were not affected in urease activity unless a chelating agent was added to the medium. As both the *ΔureTp *and *ΔnikO *mutants show lower urease activity than the wild type when grown in standard medium, we concluded that NikKMLQO is the main nickel transport system in *Brucella*. *B. suis nikA *mutants have an intact NikKMLQO nickel transporter, whose function can override the *nikA *mutation. In *B. abortus *2308 by contrast, the single *nikO *mutation produced a significant decrease in urease activity. Sequence analysis reveals that the three *B. abortus *strains sequenced so far are natural *nikA *mutants, explaining why the *nikO *mutation caused such a decrease in urease activity. When supplemented with 500 mM NiCl_2_, *B. abortus *2308 showed an increased urease activity, which probably reflects that the nickel content is not optimal in *B. abortus *and that this could be one of the factors that determines a lower urease activity in *B. abortus *when compared to *B. suis*.

*Brucella *possesses several genetic resources to cope with its needs of urease. At least three loci, *nik*, *ure1 *and *ure2 *play a role in this function. There are also some additional genes, like *cobT*, that contribute in a yet unknown way to the overall urease activity [1]. As a conclusion, *Brucella *spp. not only has at least one active urease, but also a specific, proton-gated urea transporter, and two nickel transport systems that contribute to the overall urease activity. While the urease structural genes and nickel transport systems affect the intrinsic urease activity, UreT would not affect it, but would be important for physiological processes such as the resistance to low acid conditions by increasing the efflux of urea into the bacteria, affecting in this way the overall urease activity, specially at low urea concentrations. These are the conditions faced by the bacteria in the gastrointestinal route, that it is been again recognized in the last years as an important route of infection in *Brucella *[1,2,23,24], reinforcing the idea that urease activity, and the acid resistance that it causes, is important in the life cycle of the bacteria.

## Methods

### Bacterial strains and growth conditions

The bacterial strains and plasmids used in this study are listed in Table 2. *B. abortus *strains were grown in *Brucella *broth (BB) or *Brucella *agar (BA) plates (Pronadisa, Spain). *Escherichia coli *strains were grown in Luria-Bertani broth (LB) or plates (LA). When required, media were supplemented with the following antibiotics: kanamycin (Km) 50 μg/ml, ampicillin (Ap) 100 μg/ml, or chloramphenicol (Cm) 25 μg/ml, or with 500 μM of NiCl_2_. Mating mixtures were plated in BA plates made selective with *Brucella *Selectavial, (BAF) (MAST Diagnostics, UK). All experiments with live *Brucella *were performed in a Biosafety Level 3 facility at the Department of Molecular Biology of the University of Cantabria.

**Table 2:** Bacterial strains and plasmids used in this study.

	Characteristics	Reference
**Strains**		

*Brucella abortus*		

2308	Virulent laboratory strain	

2308*ΔureTp*	2308 *ureT *polar mutant	This work

2308*ΔureT*	2308 *ureT *non-polar mutant	This work

2308*ΔnikO*	2308 *nikO *non-polar mutant	This work

*Escherichia coli*		

DH5α	Standard *E. coli *cloning strain	[36]

S17-1 λ *pir*	Mobilizing donor for conjugation	[37]

**Plasmids**		

pGEM-T Easy	PCR cloning vector	Promega

pRH016	Gateway shuttle vector	[31]

pDS132	Suicide mobilizable plasmid	[38]

pBBR1 MCS	Broad-host-range plasmid	[39]

pFJS225	U_*ureT *in pGEMT-Easy	This work

pFJS226	D_*ureT *in pGEMT-Easy	This work

pFJS227b	*ΔureT::aph *in pDS132	This work

pFJS235	pUC4K with *aphT*	This work

pFJS236	*ΔureT *in pGEMT-Easy	This work

pFJS237	*ΔnikO *in pGEMT-Easy	This work

pFJS238	*ΔureT::aphT *in pGEMT-Easy	This work

pFJS239	*ΔnikO::aphT *in pGEMT-Easy	This work

pFJS241b	*ΔureT::aphT *in pDS132	This work

pFJS242b	*ΔnikO::aphT *in pDS132	This work

pFJS243	*ureT *in pRH016	This work

pFJS244	*nikO *in pGEMT-Easy	This work

pFJS245	*nikO *in pBBR1 MCS	This work

### Molecular techniques

DNA manipulations were performed according to standard techniques [25]. Restriction enzymes were purchased from Fermentas, and primers were purchased from Sigma-Aldrich. DNA fragments were amplified by PCR from *B. abortus *2308 genomic DNA extracted as previously described [26]. High-fidelity PCR was performed using *Vent *polymerase (New England Biolabs), and standard PCR was performed using Taq (Qiagen). PCR products were purified using GenElute™ PCR Clean-Up (Sigma). Amplified products were cloned in pGEM^®^-T Easy (Promega) or pJET1.2 (Fermentas) depending on the polymerase used. The DNA sequence of the final plasmids was determined to rule out mutations introduced by PCR. Gateway cloning was made according to the manufacturer instructions (Invitrogen). The oligonucleotides used are listed in Table 1.

### Construction of an *aphT *resistance cassette

Plasmid pFJS235 carrying the aminoglycoside 3'-phosphotransferase gene (which encodes for kanamycin resistance) devoid of its transcription terminator (*aphT*) was constructed as follows. Primer *aphT*.F, derived from pUC4K [27] and located 5' from the *aph *gene, and primer *aphT*.R, derived from the *aph *sequence [28], were used to amplify a 1,005 bp DNA fragment from plasmid pUC4K. The amplified fragment was digested with *Pst*I and cloned into pUC4K/*Pst*I, yielding plasmid pFJS235. The *aphT *gene can be retrieved from pFJS235 by using *Pst*I, *Hin*cII, *Sal*I, or *Eco*RI.

### Construction of mutants and complementation plasmids

To construct a polar Δ*ureT *mutant (Δ*ureTp*) from *B. abortus *strain 2308, *ureT *was replaced by *aph*. DNA fragments both upstream and downstream of *ureT *were amplified with the following set of primers: U_BMEI0642_*Xba*I.F and U_BMEI0642_*Bam*HI.R were used to amplify a region of 578 bp upstream of *ureT *(U_*ureT*) and D_BMEI0642_*Bgl*II.F and D_BMEI0642_*Pst*I.R were used to amplify a region of 589 downstream of *ureT *(D_*ureT*). PCR fragments of the expected size were gel-purified and cloned into pGEM^®^-T Easy resulting in plasmids pFJS225 and pFJS226 respectively. pFJS225 was linearized with *Bam*HI and pFJS226 with *Bgl*II, and ligated to a 1.2 kb *Bam*HI fragment from pUC4K, containing *aph *with its transcription terminator. An *Xba*I &*Pst*I fragment of 1.4 kb was obtained directly from the partially digested ligation mixture, and cloned into pDS132 digested with *Pst*I and partially with *Xba*I, to obtain pFJS227b, that was used to construct the corresponding Δ*ureTp *mutants in *Brucella*, as described below.

For the construction of a non-polar Δ*ureT *mutant from *B. abortus *2308, two PCR fragments of 578 bp and 619 bp were generated immediately upstream and downstream of the *ureT *gene with oligonucleotides U_BMEI0642_*Xba*I.F and U_BMEI0642_*Bam*HI.R and oligonucleotides D_BMEI0642.F and D_BMEI0642_*Pst*I.R respectively. The reaction conditions for both PCRs were 30 cycles at 55°C, and 45 seconds at 72°C, using Vent polymerase. Both fragments (containing complementary regions) were ligated by overlapping PCR using oligonucleotides U_BMEI0642_*Xba*I.F and D_BMEI0642_*Pst*I.R and Taq polymerase from Qiagen, for 25 cycles at 55°C and extension time of 1 minute at 72°C. The resulting fragment containing the *ureT *deletion allele was gel-purified and cloned into pGEM^®^-T Easy to obtain pFJS236. A *Bam*HI fragment from pFJS235 containing *aphT *was introduced into the *Bam*HI site of pFJS236, resulting in plasmid pFJS238. An *Xba*I & PstI fragment from this plasmid containing the replaced *ureT *gene was cloned into pDS132 digested with *Pst*I and partially with *Xba*I, resulting in plasmid pFJS241b, that was used to create the corresponding *Brucella *mutant as described below.

For the construction of a Δ*nikO *non-polar mutant, two PCR fragments of 501 bp and 499 bp were generated immediately upstream and downstream of the *nikO *gene with oligonucleotides BAB1_1388_*Xba*I.F and RT_BAB1_1388.R, and oligonucleotides BAB1_1388_*Bgl*II.F and BAB1_1388_*Pst*I.R respectively, using Vent polymerase. Both fragments (containing complementary regions) were ligated by overlapping PCR using oligonucleotides BAB1_1388_*Xba*I.F and BAB1_1388_*Pst*I.R and Taq polymerase, and the resulting fragment containing the deleted *nikO *allele was cloned into pGEM^®^-T Easy (pFJS237). A *Bam*HI fragment from pFJS235 containing *aphT *was introduced into the *Bgl*II site of pFJS237, resulting in plasmid pFJS239. An *Xba*I &*Pst*I fragment from this plasmid containing the replaced *nikO *gene was cloned into pDS132 digested with *Pst*I and partially with *Xba*I, resulting in plasmid pFJS242b, that was used to create the corresponding *Brucella *mutant as described below.

To construct the different mutants, replacement plasmids were transformed into *E. coli *S17-1 λ *pir*, and mobilized to the corresponding *Brucella *recipient strain, by mixing equal volumes (100 μl) of liquid cultures of both donor and recipient cells on a 0.22-μm-pore-size filter. The filter was left for 4 h on a BA plate without antibiotics, soaked in PBS, and then different dilutions were plated onto BAF plates containing Cm and Km. Colonies growing in this medium represented single-crossover events. Five colonies of each construct were pooled and grown in BB, and 10^8 ^CFU were plated on BA containing 5% sucrose to select for the double crossover. Sucrose-resistant colonies were replicated in BA Cm plates, and CmS colonies were selected and analyzed by PCR and southern blot to ensure that the right mutant had been constructed.

To complement the different mutants complementation plasmids were constructed as follows: *ureT *was cloned by using the Gateway recombination cloning technology (Invitrogen) [29]. The entry vector was obtained from the *Brucella *ORFeome generated previously [30]. The destination vector, pRH016 [31], carries a chloramphenicol resistance marker, and the toxic cassette is flanked by *attR1 *and *attR2 *recombinational sites. The recombinational cloning procedure was performed as recommended by the manufacturer, to produce pFJS243. *nikO *was amplified by PCR with oligonucleotides *nikO*_*Sal*I.F and *nikO*_*Pst*I.R, cloned into pGEM^®^-T Easy to obtain pFJS244, and then subcloned into pBBR1 MCS/*Sal*I &*Pst*I to give pFJS245. Both pFJS243 and pFJS245 were transformed into *E. coli *S17-1 λ *pir *to be mobilized to *Brucella*. Complemented strains were selected in BAF Cm.

### *In vitro *susceptibility of *Brucella *to acid pH

*B. abortus *strains were grown in BB until the end of the exponential phase, washed in sterile water and resuspended at a concentration of 10^8 ^CFU/ml in citrate buffer pH 2.0 for 30 min in the presence or absence of different concentrations of urea. Bacteria were washed three times in phosphate-buffered saline (PBS), and survivors counted after dilution and plating.

### Measurement of urease activity

Urease activity was determined by measuring the amount of ammonia released from urea. Exponential cultures of bacteria grown in BB, supplemented or not with 500 μM of NiCl_2 _as indicated, were recovered by centrifugation, washed, and resuspended in PBS to a concentration of 10^8 ^CFU/ml. The preparations were then lysed using three 10-s cycles with a FastPrep system (Bio 101, Vista, CA) at the maximum setting, cooled on ice, and centrifuged for 5 min at 25,000 × g at 4°C to remove the cell debris. Crude extracts were stored at -80°C until they were used. For standard urease reactions, 5 to 10 μl of extract were added to a tube containing 200 μl of 50 mM urea in PBS and incubated for 5 min at 37°C. Urease activitiy was also measured in intact cells, in this case the pelleted bacteria were resuspended in 200 μl of either PBS (pH 7.7) or citrate buffer at different pH (3.8, 4.2, 4.6, 5.0, 5.4, 5.8, and 6.2), supplemented or not with urea at different concentrations (0, 1, 5, 10, 20, 30, 40, 50, 75, and 100 mM), and incubated at 37°C for 1 hour. The amount of ammonia released from urea hydrolysis was determined colorimetrically by the modified Berthelot reaction [32], and the total protein concentration was measured by a Bradford assay [33]. Urease specific activity was expressed in μmol of NH3 min^-1^mg^-1 ^protein (for crude extracts) and pmol of NH_3 _min^-1 ^log10 cfu^-1 ^(for intact cells).

### RNA isolation and reverse transcriptase PCR (RT-PCR)

3 ml of a bacterial culture in mid-log phase (OD_600 _= 0.6-0.7) were stabilized with RNAprotect Bacteria Reagent (Qiagen). After harvesting the cells, they were resuspended in 300 μl of TE containing lysozyme 1 mg/ml, and incubated for 15 min at room temperature. They were treated with 15 μl of 10% Zwittergent 3-16 (Calbiochem) and 6 μl of 20 mg/ml of proteinase K for 1 h at 37°C, and then total RNA was extracted with the RNeasy Mini System (Qiagen) in combination with the RNase-Free DNase Set (Qiagen). cDNA was generated by using Superscript III RT (Invitrogen) according to the manufacturer's protocol. 1 μl of the resulting cDNA was used for each PCR. As a negative control, reactions were also run on RNA templates without RT treatment, and as a positive control, each reaction was also made with purified genomic DNA as template. The cycling parameters were 30 cycles of 94°C for 30 s, 55°C for 30 s, and 72°C for 1.5 min. The resulting amplicons were analyzed in 0.8% agarose gels. Primers were designed with Primer3 software [34].

### Genomic data and analysis

The complete genome sequence and annotation of the *B. abortus *2308 strain was obtained fron GenBank (Accession numbers AM040264 and AM040265 for chromosomes I and II respectively).

Blast comparisons against the microbial genome database were performed via web at the NCBI Blast server [35].

### Statistical analysis

A statistical analysis was performed using Prism3, version 3.0(GraphPad Software, San Diego, CA). Statistical significance wascalculated using either a nonparametric Mann-Whitney test or an unpaired *t *test. A *P *value of < 0.05 was considered statistically significant.

## Authors' contributions

FJS designed and supervised the work and wrote the paper. AC performed all the microbiological work and the different urease activity assays. AS did the transcriptional analysis of the urease operon. JMGL performed the genomic analysis and bioinformatic work and also wrote the paper.
